# One-Pot Synthesis of Cu_2_ZnSnSe_4_ Nanoplates and their Visible-Light-Driven Photocatalytic Activity

**DOI:** 10.1186/s11671-017-2428-7

**Published:** 2018-01-10

**Authors:** Zhenzhen Han, Nan Li, Aihua Shi, Haohua Wang, Feng Ma, Yi Lv, Rongqian Wu

**Affiliations:** Shaanxi Provincial Center for Regenerative Medicine and Surgical Engineering, Institute of Advanced Surgical Technology and Engineering, First Affiliated Hospital, Xi’an Jiaotong University, 76 West Yanta Road, P.O. Box 124, Xi’an, Shaanxi province 710061 China

**Keywords:** Cu_2_ZnSnSe_4_ nanoplates, One-pot thermal chemical method, Photocatalysis

## Abstract

A SeO_2_ ethanol solution as the facile precursor has been used for the preparation of quaternary Cu_2_ZnSnSe_4_ (CZTSe) nanoplates. Monodispersed single-phase CZTSe nanoplates have been prepared successfully by a facile one-pot thermal chemical method. The as-prepared CZTSe nanoplates show uniform morphology with a bandgap of ~ 1.4 eV. As a proof of concept, the CZTSe nanoplates have been used as a visible-light-driven photocatalyst for Rhodamine B dye degradation and show high photocatalytic activity and stability. The excellent dye removal is mainly ascribed to the efficient light utilization of CZTSe nanoplates.

## Background

Chemical pollutants in natural water have aroused extensive attention due to its serious damage to the environment, and photocatalytic degradation technique based on semiconductors by employing solar energy has been considered as a promising solution to this problem [1]. However, typical photocatalysts, such as TiO_2_ and ZnO, can only absorb ultraviolet (UV) light. In fact, about 50% solar energy is mainly concentrated in the visible light region while UV light accounts for less than 4% of the solar spectrum [2]. To utilize visible light and improve the photocatalytic activity, various efficient photocatalysts have been explored and applied in organic pigment degradation, water splitting, and solar cell absorbers [3]. Among several photocatalysts, copper-based ternary and quaternary chalcogenide semiconductors, such as Cu_2_SnS_3_, CuIn_x_Ga_1-x_Se_2_, and Cu_2_ZnSnS_4_, have been of broad interest owing to their outstanding optoelectronic properties with large absorption coefficient (> 10^4^ cm^−1^), good stability, and suitable bandgap energy (1.0–1.5 eV) [4–9].

Cu_2_ZnSnSe_4_ (CZTSe) nanocrystals and thin films with inexpensive, non-toxic, and earth-abundant constituent elements have been investigated extensively in recent years [8, 10–15]; however, there are a few reports related to the study of nanoplate morphology [16, 17]. Hot-injection and one-pot thermal chemical method are usually applied to the synthesis the CZTSe nanostructures [18–21]. However, the Se precursors used in these methods are expensive, toxic, or unstable. Herein, a facile Se precursor which dissolves SeO_2_ powder in ethanol is developed in this study.

Here, we report a one-pot thermal chemical method of CZTSe nanoplate synthesis using a facile Se precursor. The visible-light-driven photocatalytic activity and recycle performance of CZTSe nanoplates were investigated. The CZTSe nanoplates have potential in wastewater treatment.

## Methods/Experimental

### Synthesis of CZTSe Nanoplates

All chemicals used in this work were purchased from Aladdin and used directly. Typically, 1.0 mmol Cu(acac)_2_, 0.5 mmol Zn(OAc)_2_·2H_2_O, 0.5 mmol SnCl_2_·2H_2_O, and 2.0 mmol SeO_2_ dissolved in 4 mL ethanol were added into 20 mL oleylamine (OLA) in a 100-mL three-neck flask. The mixture was degassed at 130 °C for 1 h, purged with Ar for 30 min, and then heated to 280 °C for 1 h. The nanoplates were washed with hexane and ethanol three times by centrifugation at 8000 rpm for 5 min. The black powder was collected and dried at 60 °C under vacuum. Before photocatalytic reaction, the nanoplates were hydrophilic treated with Na_2_S to remove the long-chain OLA ligands [8].

### Characterizations

Powder X-ray diffraction (XRD, D/max 2200, Rigaku, Japan) using Cu Kα radiation (40 kV, 100 mA) and a Raman spectrometer (Inviareflex, Renishaw, UK) coupled with a 514 nm laser were applied to analyze the phase of the samples. Transmission electron microscopy (TEM, JEM-2100F, JEOL., Japan) and scanning electron microscopy (SEM, Quatan 250FEG, FEI, USA) measurements were performed to characterize the morphologies of the samples. The UV-vis absorption spectra of CZTSe nanoplate powder and Rhodamine B (RhB) aqueous solution were recorded on a UV/vis spectrometer by using the integrating sphere and cuvette, respectively (Lambda, Perkin Elmer, USA).

### Photocatalytic Activity Measurements

Visible-light-driven photocatalytic activity of the CZTSe nanoplates was evaluated by photodegradation of the RhB aqueous solution (10 mg/L) at ambient temperature. A 300-W Xe lamp equipped with a 420 nm cutoff filter was used as a visible light source. Typically, 50 mg photocatalyst was added into 100 mL of RhB aqueous solution. The solution was continuously stirred in the dark for 12 h to ensure the adsorption-desorption equilibrium before irradiation. The concentration of the residual RhB was monitored at a sequence of time intervals by the UV-vis spectrometer at 554 nm to calculate the degradation rate based on the Beer-Lambert Law.

## Results and Discussion

**Fig. 1:**
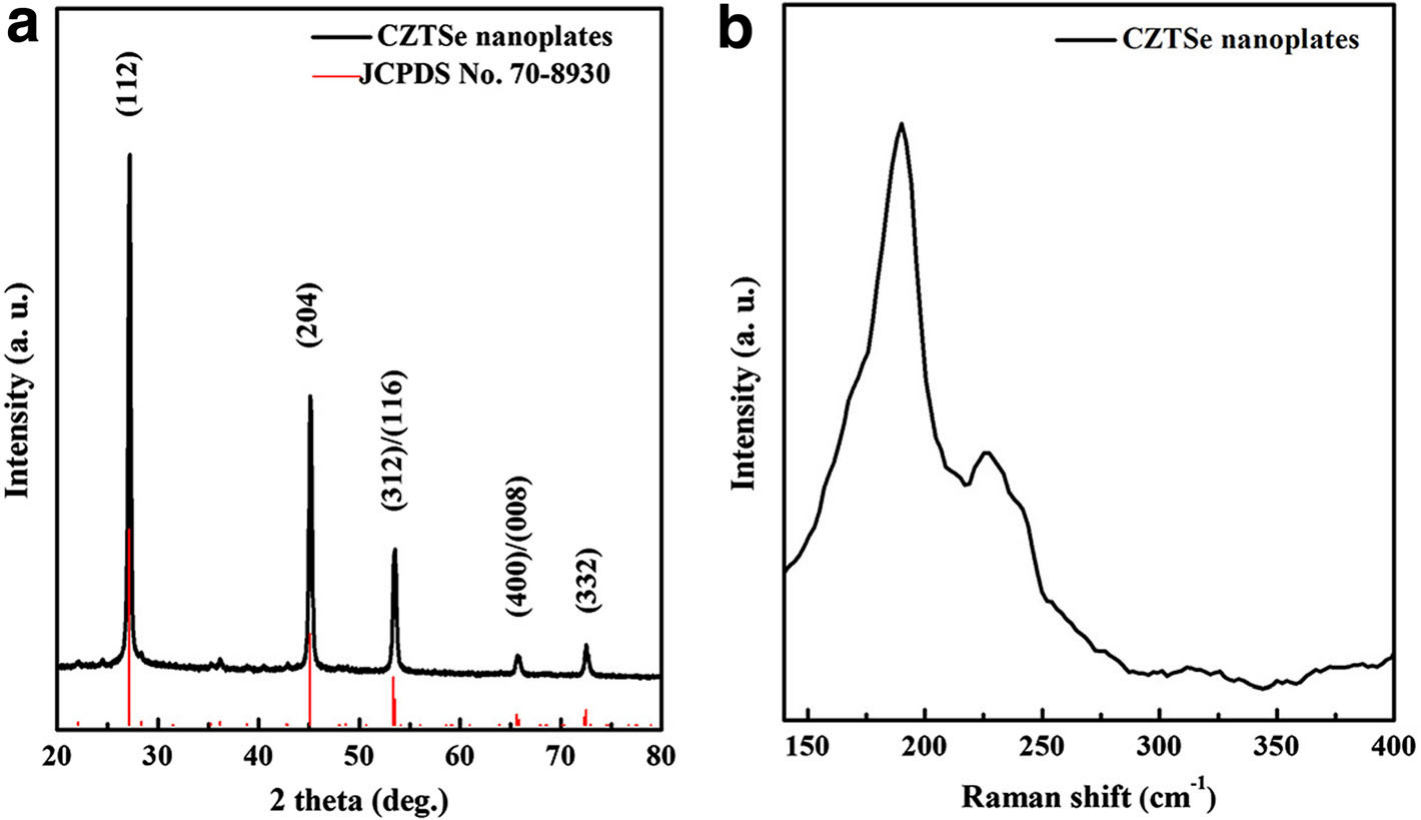
**a** XRD pattern and **b** Raman spectrum of CZTSe nanoplates

In Fig. 1a, all diffraction peaks of the as-prepared CZTSe sample in XRD pattern can be clearly ascribed to the tetragonal kesterite structure of Cu_2_ZnSnSe_4_ (JCPDS No. 70-8930). The diffraction peaks at 27.1°, 45.1°, 53.5°, 65.8°, and 72.5° can be indexed to (112), (204), (312)/(116), (400)/(008), and (332) of CZTSe, respectively. Raman scattering was further applied to confirm the pure phase, as shown in Fig. 1b. Three peaks in Raman spectrum also verify the pure phase of the CZTSe nanoplates, and no other binary phases of Cu_x_Se and ZnSe (main peaks at 262 and 252 cm^−1^, respectively) and ternary phase of Cu_2_SnSe_3_ (main peak at 180 cm^−1^) are observed. Therefore, neither XRD nor Raman result reveals any secondary phase, suggesting the pure quaternary phase of CZTSe nanoplates.

**Fig. 2:**
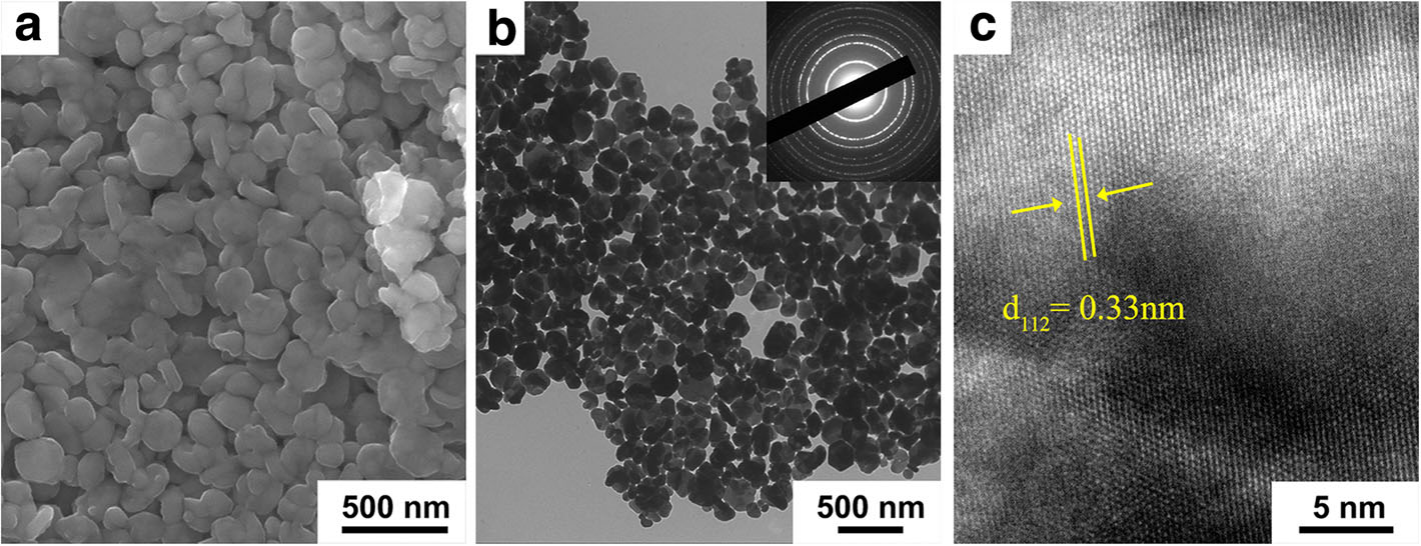
**a** SEM image. **b** TEM image (inset: SAED pattern). **c** HRTEM image of CZTSe nanoplates

Figure 2 shows the SEM, TEM, and high-resolution TEM (HRTEM) images of the as-synthesized CZTSe nanoplates. It can be observed in Fig. 2a that the CZTSe samples have plate-like and uniform morphology. The average size of CZTSe nanoplates calculated from Fig. 2b is ~ 210 nm, which matches well with the SEM observation. The selected area electron diffraction pattern (SAED) shown in the inset of Fig. 2b indicates high crystallization of the nanoplates. Figure 2c exhibits the HRTEM image of a nanoplate, displaying its primarily ordered crystalline structure and 0.33-nm interplanar d-spacing indexed to the (112) of CZTSe.

**Fig. 3:**
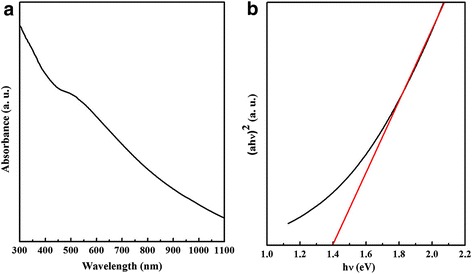
**a** UV-vis absorption spectrum and **b** bandgap of CZTSe nanoplates

UV-vis absorption spectrum reveals the optical property of CZTSe nanoplates. It can be seen from Fig. 3a that the CZTSe nanoplates have entire visible light region absorbing performance. The bandgap can be calculated from the equation: *αhν* = *A*(*hν−E*_g_)^1/2^, where *A*, *α*, *h*, *v*, and *E*_g_ are a constant, the absorption coefficient, plank constant, light frequency, and bandgap, respectively. The bandgap of CZTSe nanoplates obtained from Fig. 3b is ~ 1.4 eV, which is a little larger than that of CZTSe bulk due to the quantum confinement effect [9].

**Fig. 4:**
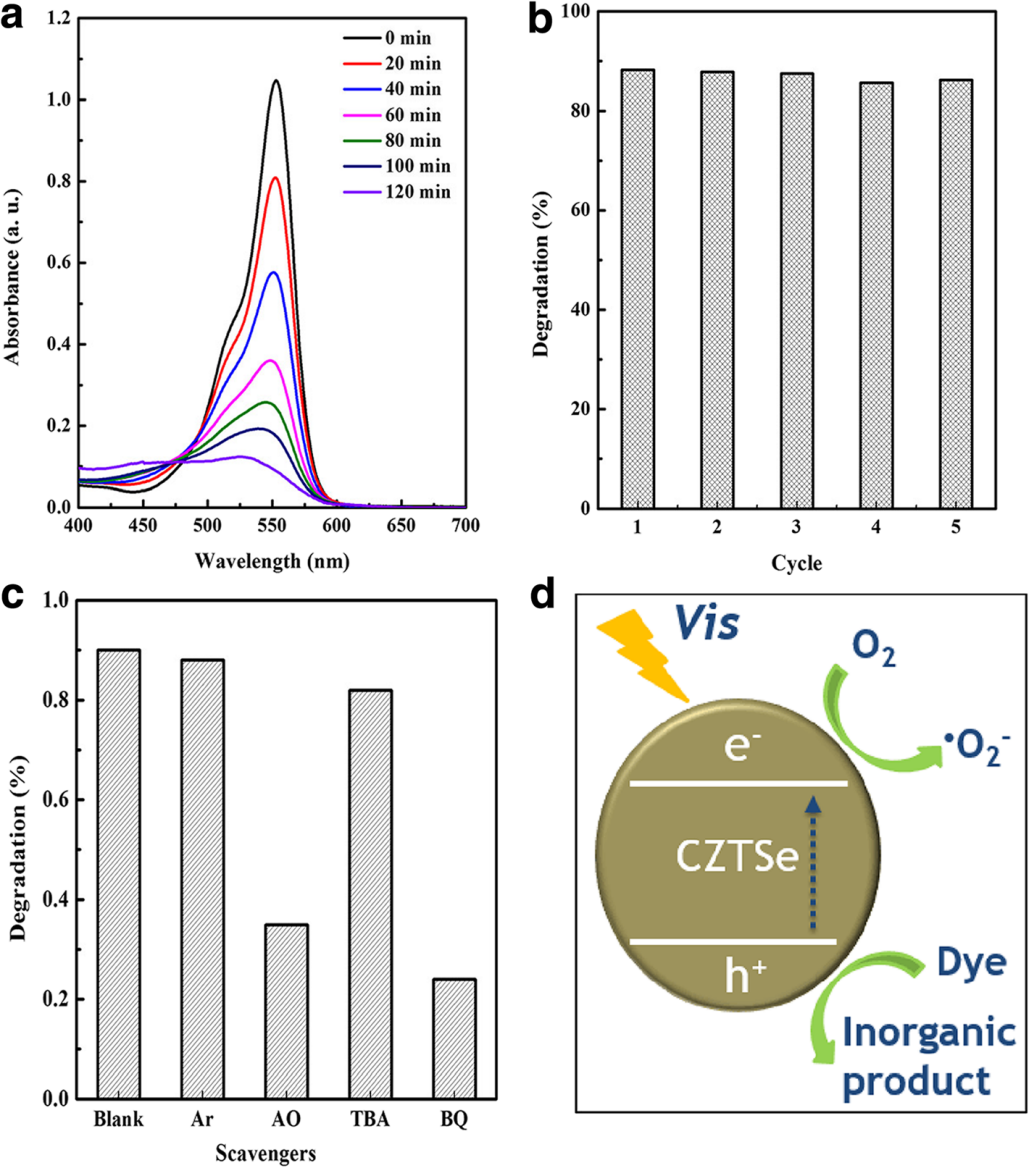
**a** RhB degradation. **b** Cycle test. **c** Effects of various quenchers on the degradation efficiency of RhB. **d** Scheme of the photocatalytic degradation process

Photocatalytic activity of as-prepared CZTSe nanoplates is evaluated with photodegradation of RhB aqueous solution under visible light region. It can be seen from Fig. 4a that ~ 90% RhB are photodegraded within 120 min. The stability and reusability of photocatalyst play a key role in the application of disintegrating ecological pollutants. Thus, five cycle experiments were carried out, and the results are shown in Fig. 4b. CZTSe nanoplates keep high photodecomposition activity in cycle tests, indicating their high stability in photocatalytic reaction. It is well known that photo-oxidation process could be mainly related to several active species, such as hydroxyl radicals (•OH), superoxide radicals (•O_2_^−^), and hole (h^+^). Because both the *E*_CB_ and *E*_VB_ of CZTSe are more negative than the standard redox potentials of *E*^θ^ (O_2_/•O_2_^−^) and *E*^θ^ (H_2_O/•OH), the •O_2_^−^ rather than •OH can be generated in the photocatalytic process. To further verify the main active species, Argon (Ar), ammonium oxalate (AO), tert-butanol (TBA), and benzoquinone (BQ) were applied for the removal of O_2_, h^+^, •OH, and •O_2_^−^, respectively. The reaction system with corresponding quenchers (0.1 mmol) was illuminated for 120 min, and the results are shown in Fig. 4c. It can be confirmed that the O_2_ is the necessity in the photo-oxidation process, rarely •OH is produced, and both the •O_2_^−^ and h^+^ are active species. The •O_2_^−^ plays a more important role than h^+^ for its sharper degradation efficiency decrease after trapping. Figure 4d shows the possible mechanism of the photocatalytic reaction process. Electrons are excited from valence band (VB) to conduction band (CB) under illumination. The photogenerated electrons are captured by O_2_ in the aqueous solution to form •O_2_^−^, which is highly oxidative and can degrade RhB into inorganic products. Simultaneously, the holes directly function as oxidants. Thus, the visible-light-driven photocatalytic activity is achieved by the full use of visible light of the CZTSe nanoplates.

## Conclusions

A SeO_2_ ethanol solution as the facile precursor has been used for the preparation of quaternary CZTSe nanoplates. Monodisperse CZTSe nanoplates have been prepared successfully by a facile one-pot thermal chemical method. As a proof of concept, the CZTSe nanoplates have been used as visible light response photocatalyst for RhB dye degradation. The efficient dye removal is mainly ascribed to the efficient light utilization of CZTSe nanoplates.
